# Stroke survivors with severe mental illness: Are they at-risk for increased non-psychiatric hospitalizations?

**DOI:** 10.1371/journal.pone.0182330

**Published:** 2017-08-11

**Authors:** Flavius Robert Lilly, Joel Culpepper, Mary Stuart, Donald Steinwachs

**Affiliations:** 1 Graduate School, University of Maryland, Baltimore, Maryland, United States of America; 2 Veterans Affairs Maryland Health Care System, Baltimore, Maryland, United States of America; 3 Department of Sociology, Anthropology, and Health Administration and Policy, University of Maryland, Baltimore County, Baltimore, Maryland, United States of America; 4 Department of Health Policy and Management, Johns Hopkins University Bloomberg School of Public Health, Baltimore, Maryland, United States of America; Yokohama City University, JAPAN

## Abstract

**Background:**

This study examined outcomes for two groups of stroke survivors treated in Veteran Health Administration (VHA) hospitals, those with a severe mental illness (SMI) and those without prior psychiatric diagnoses, to examine risk of non-psychiatric medical hospitalizations over five years after initial stroke.

**Methods:**

This retrospective cohort study included 523 veterans who survived an initial stroke hospitalization in a VHA medical center during fiscal year 2003. The survivors were followed using administrative data documenting inpatient stroke treatment, patient demographics, disease comorbidities, and VHA hospital admissions. Multivariate Poisson regression was used to examine the relationship between patients with and without SMI diagnosis preceding the stroke and their experience with non-psychiatric medical hospitalizations after the stroke.

**Results:**

The study included 100 patients with SMI and 423 without SMI. Unadjusted means for pre-stroke non-psychiatric hospitalizations were higher (p = 0.0004) among SMI patients (1.47 ± 0.51) compared to those without SMI (1.00 ± 1.33), a difference which persisted through the first year post-stroke (SMI: 2.33 ± 2.46; No SMI: 1.74 ± 1.86; p = 0.0004). Number of non-psychiatric hospitalizations were not significantly different between the two groups after adjustment for patient sociodemographic, comorbidity, length of stay and inpatient stroke treatment characteristics. Antithrombotic medications significantly lowered risk (OR = 0.61; 95% CI: 0.49–0.73) for stroke-related readmission within 30 days of discharge.

**Conclusions:**

No significant differences in medical hospitalizations were present after adjusting for comorbid and sociodemographic characteristics between SMI and non-SMI stroke patients in the five-year follow-up. However, unadjusted results continue to draw attention to disparities, with SMI patients experiencing more non-psychiatric hospitalizations both prior to and up to one year after their initial stroke. Additionally, stroke survivors discharged on antithrombotic medications were at lower risk of re-admission within 30 days suggesting the VHA should continue to focus on effective stroke management irrespective of SMI.

## Introduction

Persons with severe mental illness (SMI) including schizophrenia, bipolar disorder and major depression are approximately twice as likely to have a stroke [1,2]. Increased stroke risk among those with SMI has been attributed to unhealthy lifestyle choices like tobacco use [3], substance abuse [4], low physical exercise [5], poor nutrition [6], and morbid obesity [7]. Use of atypical antipsychotics, such as olanzapine and clozapine, also account for significant weight gain and may contribute to risk for stroke [7,8]. Additionally, functional impairments associated with SMI place them among the most vulnerable of social groups where they encounter poverty, chronic unemployment, substandard housing, poorer access to quality health care, disrupted social relations, and delayed preventative care, which exacerbate overall risk for poor health outcomes [6, 8–11]. Consequently, individuals with SMI experience about 25 years of reduced life expectancy [12].

Little is known about stroke survivors with SMI regarding their patterns of post-stroke hospitalization. Unplanned hospitalizations among stroke survivors are costly [13], may reflect suboptimal patient outcomes [14], and are often related to other co-morbid conditions. However, there is conflicting information on whether persons with SMI are at increased risk for non-psychiatric hospitalization after an initial stroke. For instance, a recent systematic review [15] of hospitalization among stroke survivors does not mention SMI as a contributing factor in any reviewed studies. Yet, previous research suggests that stroke survivors with SMI may be particularly vulnerable to poor stroke outcomes requiring hospitalization because they have high readmission rates across a spectrum of other health conditions, even after adjusting for other comorbidities and lifestyle factors [11]. Factors that have been related to post-stroke hospitalizations among stroke survivors include age [16], race [17], education [18], insurance coverage [19], history of physical comorbidity prior to stroke [20], hospitalizations prior to stroke [20], hypertension [17], coronary artery disease [21], diabetes [21], and residential proximity to hospital [22].

The current study compares the risk of non-psychiatric hospitalizations for veteran stroke survivors with and without SMI and examines patterns of hospitalization in Veterans Health Administration (VHA) hospitals over a five-year period. Veteran stroke survivors, whether or not they have severe mental illnesses, are vulnerable and have relatively poor health status. For example, the health status of veterans is consistently found to be poorer in comparison to the rest of the United States with increased rates of obesity [23], diabetes [24], homelessness [25], and substance abuse [26]. Individuals with SMI in the general population who have never had a stroke have higher non-psychiatric hospitalizations even after adjusting for other co-morbidities and lifestyle factors [11]. Yet, there is a paucity of research on the influence of SMI on non-psychiatric hospitalizations among veteran stroke survivors receiving care in the VHA system. It is notable that in a relatively recent systematic review [15] of predictors of hospital admissions after stroke that not a single reviewed study considered SMI as either a primary independent variable or as a covariate related to post-stroke hospitalizations. The aim of this study is to begin to fill the gap in the literature on the role of SMI in post-stroke non-psychiatric hospitalizations.

## Materials and methods

### Hypothesis

We conducted a five-year retrospective cohort study of veterans identified through the U.S. Department of Veterans Integrated Service Network Five [27] as having an index stroke in 2003 and receiving their care in the VHA system.

Our hypothesis was that stroke survivors with SMI would have increased risk of non-psychiatric hospitalizations over five-years compared to stroke survivors without SMI after adjustment for inpatient stroke treatment variation, age, race, marital status, education, income, health insurance coverage above VHA benefits, proximity of patient’s home to a VHA hospital, physical co-morbid conditions, prior pre-stork hospitalizations, mental co-morbid conditions and history of stroke risk factors of hypertension, diabetes, and peripheral vascular disease. The rationale for this hypothesis was that individuals with SMI have myriad lifestyle factors, health care access difficulties, and medication side effects, which increase their risk of physical diseases resulting in greater non-psychiatric hospitalization rates.

### Defining stroke, non-psychiatric hospitalizations and SMI

The International Classification of Diseases, Ninth Revision (ICD-9) was used to identify ischemic and hemorrhagic stroke participants. ICD-9 diagnosis codes have been extensively used for stroke outcomes research [15, 28] and have been validated with high sensitivity (86%), specificity (95%), and positive predictive value (86–92%) [29]. The following ICD-9 CM diagnostic codes were used to identify stroke patients from either inpatient or outpatient encounters: 433.x1, 434 (excluding 434.x0), or 436. Outpatient records were important to consider because many VHA medical centers contract with their affiliated medical school hospital to provide specialty care services like acute stroke care. Thus, a majority of acute stroke care is not provided in the VHA facility. Once a patient has been stabilized they may be transferred to the VHA medical center or to some other level of rehab or nursing care. Thus, an outpatient claim may be the first instance that the stroke event gets captured within the claims system. Outpatient claims were used to capture those patients whose index stroke hospitalization occurred in a non-VA facility. Additionally, ICD-9 code 436 is not routinely used to identify stroke, however, after consulting with stroke specialists at the VHA, it was recommended for inclusion because it was a code commonly used to capture strokes in patients for whom they personally did not provide care. Moreover, based on previous work in identifying neurologic cohorts from VHA data, the stroke diagnostic codes must occur as the first or second diagnosis indicating a primary diagnosis. The VHA captures up to ten diagnosis codes per encounter with codes listed in descending order of their impact on that episode of care. This method identified a total of 2,299 veterans. Nearly 80% of those identified based on an inpatient encounter had diagnosis codes of 431–434, whereas 89% of those identified based on an outpatient encounter had only a ICD-9 436 diagnostic code. Thus, ICD-9 code 436 was used when there was unambiguous evidence of stroke but that index event (acute care inpatient encounter) was not managed in the VHA facility. Finally, we then looked back three years for the presence of a stroke diagnosis to identify only veterans with an index stroke hospitalization in 2003 yielding 523 such cases who comprise the study group.

Non-psychiatric hospitalizations were defined as inpatient admissions for any cause except treatment for psychiatric or substance abuse disorders from the VHA Inpatient data file. Consistent with previous research, non-psychiatric hospitalizations were considered within the following time intervals from index stroke discharge: within 30 days, 31 to 90 days, 91 to 364 days, and within year-long intervals thereafter up to five years [30]. All admissions within 30-days post-stroke discharge were due to stroke-related complications. Causes of hospitalization were determined by examining the principle ICD-9 diagnosis code for each hospitalization. ICD-9 codes were further categorized into eleven discrete groupings, including: neurologic, cardiovascular, orthopedic, infections, kidney disorders, endocrine disorders, digestive disorders, cancer, blood disorders, respiratory disorders, or miscellaneous. SMI was defined in our sample by ICD-9 codes for schizophrenia (295.00–295.05; 295.10–295.15; 295.20–295.25; 295.30–295.35; 295.40–295.45; 295.50–295.55; 295.60–295.65; 295.70–295.75; 295.80–295.85; and 295.90–295.95), bipolar disorder (296.00–296.06; 296.10–296.16; 296.40–296.46; 296.50–296.56; 296.60–296.66; 296.70; 296.80–296.82; 296.89–296.90; and 296.81), and major depression (296.20–296.26 and 296.30–296.36) [31]. ICD-9 codes 297, 298, and 300 were also initially included in our determination of SMI, however, none of these codes were present in the administrative data over the two-year look-back period. Likewise, A patient was categorized as having SMI if at least one instance of an ICD-9 code occurred in the two-year look-back period in either inpatient or outpatient diagnostic fields. Randomly selected medical charts were reviewed (n = 20) to validate agreement with this classification algorithm. In this review, every patient categorized as having SMI was found to have had more than three previous encounters with the VHA related to SMI.

### Study covariates

Table 1 defines the covariates, their central tendency, and coding scheme. Demographic covariates included age (18–44, 45–64, 65–74, or >75 years), marital status (married or not married), annual income (in 2002 dollars), health insurance coverage (dual beneficiary or only VHA benefits), and proximity of patient’s home to a VA hospital (in miles). Race was coded dichotomously (Caucasian or non-Caucasian) because 97.8% of non-whites were African American. Sex was excluded as a covariate because only 3% of the sample were women. History of physical comorbidity was measured as a composite score using the Elixhauser Index [32], which is a method of categorizing 30 comorbidities of patients based on the ICD-9 diagnosis codes found in administrative data. History of mental health comorbidity was measured using ICD-9 codes for post-traumatic stress disorder (309.81), acute depression (311; 308; 309.0; 309.1; and 313.1) and substance abuse (291; 292, 303; 304; 305; 535.3; 571.1; 648.3; and 790.3). History of stroke risk factors was measured by presence of ICD-9 codes for hypertension (401.x; 402.x; 403.x; and 405.x), diabetes (250.0x–250.4x; 250.7x; and 250.9x), and peripheral vascular disease (440.x; 441.x; 443.1; 443.9; 447.1; 557.1; 557.9; and V43.4) calculated for the year prior to stroke. Counts of 1-year pre-stroke non-psychiatric hospitalizations may be predictive of future hospitalizations and were considered as a covariate [33]. Length of hospital stay at index stroke was measured in days. Quality of stroke treatment was measured using three of ten indicators identified by the Joint Commission [34] as evidence for appropriate stroke care. These indicators included: appropriate assessment for rehabilitation (yes or no), appropriately discharged on a statin (yes or no), and appropriately discharged on antithrombotic medication (yes or no). SAS algorithms (Appendix 1) were written to identify ICD-9 diagnosis, procedure, and inpatient pharmacy administration codes indicative of the Joint Commission stroke processes of care.

**Table 1:** Description Independent Variables in the study population (N = 523).

Study Variables	Central Tendency ^a^^,^ ^b^	Measurement and Coding
History of Severe Mental Illness: Yes	19.1% (n = 100)	1 = Yes or 0 = No
History of Substance Abuse: Yes	19.9% (n = 104)	1 = Yes or 0 = No
History of Acute Depression: Yes	22.9% (n = 120)	1 = Yes or 0 = No
History of PTSD: Yes	42.9% (n = 224)	1 = Yes or 0 = No
History of Hypertension: Yes	84.5% (n = 441)	1 = Yes or 0 = No
History of Diabetes: Yes	43.1% (n = 225)	1 = Yes or 0 = No
History of PVD: Yes	14.8% (n = 77)	1 = Yes or 0 = No
Race: Not Caucasian	56.1% (n = 277)	1 = Not Caucasian or 0 = Caucasian
Age at Stroke: 18–44 years	5.5% (n = 29)	1 = 18–44 years
45–64 years	38.4% (n = 201)	2 = 45–64 years
65–74 years	26.6% (n = 139)	3 = 65–74 years
>75 years	29.5% (n = 154)	4 = >75 years
Marital Status: Married	41.3% (n = 216)	1 = married or 0 = Not married
Discharge Antithrombotic Therapy: Yes	91.0% (n = 476)	1 = Yes or 0 = No
Rehabilitation Assessment: Yes	18.9% (n = 99)	1 = Yes or 0 = No
Lipid Management (Statin): Yes	44.4% (n = 232)	1 = Yes or 0 = No
Insurance Coverage: Dual	43.3% (n = 226)	1 = ≥ Dual Beneficiary or 0 = Only VHA Benefits
Income	$\bar{X}$ = $15,811 [0–291,745]	Annual income in dollars for the year 2002
Proximity to Hospital	$\bar{X}$ = 11.9 miles [0–117]	Miles from patient’s residence to VHA Hospital
Hospitalization: Pre-Stroke < 1 Year ^c^	$\bar{X}$ = 1.09 admits [0–13 admits]	Any hospital admission in the previous year before initial stroke excluding hospitalizations where the primary ICD-9 discharge diagnosis was a mental health disorder, or substance abuse disorder
Length of Stay for Index Stroke	Median = 8 days [1–55 days]	Length of first hospital stay for index stroke measured in days

a) This column displays either percent (n) or mean [range] of the study population for each variable.

b) Certain processes of care were found to be undetectable using administrative data and are not presented in this chart, including antithrombotic therapy by hospital day 2, t-PA administration, and ambulation by day 2.

c) Pre-stroke < 1year was determined individually for each patient by looking back 365 days from the date of initial stroke.

### Statistical methods

Unadjusted bivariate analyses were used to describe the differences between stroke survivors with SMI compared to those without SMI for all covariates and hospitalizations over time. Bonferroni corrected significance was used to minimize the chances of making a Type I error across multiple comparisons with a p< 0.0030 needed for statistical significance. Hospitalizations in the VHA dataset were skewed to the right with substantial zero admissions at each time interval and had distributions with long right tails. Deviance and Kolmogorov-Smirnov tests for goodness-of-fit were calculated for negative binomial, zero inflated negative binomnial, ordered probit and logit, and Poisson distribution models for each time period examined. The data best fit a Poisson distribution model for every time period except for between 4 and 5 years post-stroke, where a negative binomial model had preferable deviance and Kolmogorov-Smirnov scores. However, the Poisson model for between 4 and 5 years post-stroke had a nearly equal mean (1.48) and variance (1.63). Therefore, for consistency Poisson regression was used at every time period with log-linked functions and corrections for overdispersion to test the hypothesis that SMI is associated with non-psychiatric hospitalizationsAdditionally, negative binomial regression model for the time period between 4 and 5 years post-stroke was calculated yielding equivalent results as the Poisson regression. Confounding between the variable SMI and other independent variables was assessed by adding and then removing each independent variable to a Poisson model. Independent variables that altered the parameter estimate for SMI by greater than ±15% were considered to be confounding and were removed from final models. All statistical calculations were performed using SAS version 9.3 [35]. The University of Maryland, Baltimore institutional review board approved the study.

## Results

The study population consisted of 523 patients with an index stroke in fiscal year 2003, 100 with SMI comorbidity and 423 with no SMI comorbidity. Table 2 summarizes the demographic, comorbid, and treatment differences between those with and without SMI. Patients with SMI comorbidity were significantly more likely to be younger and without a marital partner, only insured by the VHA, have more comorbid diagnoses, have more pre-stroke non-psychiatric hospitalizations and peripheral vascular disease. Patients with SMI did not significantly differ from those without SMI by race, proximity to hospital, income, history of hypertension or diabetes, hospital length of stay, or by any of the quality of stroke care indicators.

**Table 2:** Baseline characteristics of the study population (N = 523) at index stroke in 2003.

	No SMI (N = 423)	SMI (N = 100)	Total	P
Age at Time of Stroke				
18–44 years	13 (3.1%)	16 (16.0%)	29 (5.5%)	<0.0001
45–64 years	146 (34.5%)	55 (55.0%)	201 (38.4%)	
65–74 years	123 (29.1%)	16 (16.0%)	139 (26.6%)	
≥ 75 years	141 (33.3%)	13 (13.0%)	154 (29.5%)	
Non-Caucasian	220 (55.6%)	57 (58.2%)	277 (56.1%)	0.6414
Married	186 (43.9%)	30 (30.0%)	216 (41.3%)	0.0107
Insurance: Only VHA	227 (54.0%)	70 (70.0%)	297 (56.8%)	0.0028
Proximity to Hospital	13.37 ± 24.01	10.18 ± 14.73	11.95 ± 17.57	0.2623
Income	$16.0k ± 23.8k	$14,6k ± 22.5k	$15.8k ± 23.5k	0.5721
Elixhauser Index Score	5.2 ± 2.15	6.9 ± 2.44	5.53 ± 2.31	<0.0001
Prior Hospitalizations	1.15 ± 2.69	1.90 ± 3.49	1.29 ± 2.87	0.0185
History of Depression	53 (12.5%)	67 (67%)	120 (22.9%)	<0.0001
History of Substance Abuse	65 (15.4%)	39 (39.0%)	104 (19.9%)	<0.0001
History of PTSD	142 (33.65)	82 (82.0%)	224 (42.8%)	<0.0001
History of Hypertension	362 (85.8%)	70 (79.0%)	432 (82.6%)	0.0921
History of Diabetes	180 (42.7%)	45 (45.05)	225 (43.0%)	0.6702
History of Vascular Disease	69 (16.4%)	8 (8.0%)	77 (14.7%)	0.0342
Appropriate Statin	189 (44.7%)	43 (43.0%)	232 (44.4%)	0.7609
Appropriate Antithrombotic	385 (91.0%)	91 (91.0%)	476 (91.0%)	0.9958
Appropriate Rehabilitation	81 (19.2%)	18 (18.0%)	99 (18.9%)	0.7920
Length of Hospital Stay	10.8 ± 16.7	11.2 ± 18.9	11.0 ± 17.4	0.6431

Baseline characteristics are represented as counts and percentages for categorical variables and by means and standard deviations for continuous variables. Chi-square analyses were utilized to detect statistically significant differences among categorical variables and T-tests were utilized to detect statistically significant differences among continuous variables.

Causes of non-psychiatric hospitalizations were examined in the year preceding stroke and after being discharged from inpatient care for index stroke. In the year prior to stroke, patients with SMI had significantly (p<0.05) more admissions related to cardiovascular (SMI 46.3% vs. No SMI 31.6%) and infectious (SMI 17.0% vs. No SMI 8.7%) causes, and fewer admissions related to orthopedic (SMI 1.4% vs. No SMI 10.1%) causes. Patients with SMI had significantly (p<0.05) more hospitalizations related to cardiovascular (SMI 42.5% vs. No SMI 31.2%) causes and significantly fewer hospitalizations related to recurrent stroke (SMI 1.5% vs. No SMI 6.7%) during the first year post-stroke. Patients with SMI, who were re-hospitalized with a stroke complication within 30 days after being discharged for index stroke had significantly more cardiovascular complications (SMI 42.8% vs. No SMI 32.4%), but fewer hospitalizations related to neurological causes (SMI 18.4% vs. 32.4%). During the period 31 to 90 days post-stroke SMI patients had significantly more hospitalizations related to cardiovascular (SMI 40.0% vs. No SMI 25.5%) and infectious (SMI 20.0% vs. No SMI 9.9%) causes. Patients with SMI had significantly more cardiovascular causes (SMI 37.0% vs. No SMI 26.5%) of hospitalization during the period of 1 to <2 years post-stroke. There were no significant differences between patients with and without SMI regarding causes of hospitalization in any time period greater than two years.

Table 3 summarizes the differences in hospitalizations for patients with and without SMI at various time intervals from index stroke. Mean pre-stroke non-psychiatric hospitalizations were higher (p = 0.0004) among patients with SMI (1.47 ± 0.51) compared to those without SMI (1.00 ± 1.33) in our sample. Mean non-psychiatric hospitalizations continued to be significantly higher among patients with SMI compared to patients without SMI at all the examined time intervals during the first year after stroke, and for the entire first year considered cumulatively. In time intervals 1 to <2 years, 2 to <3 years, and 3 to <4 years post-stroke there was no statistical difference between mean non-psychiatric hospitalizations among patients with and without SMI. However, by the fourth year after stroke, patients with SMI have significantly fewer non-psychiatric hospitalizations compared to those without SMI.

**Table 3:** Comparison of number and average of non-psychiatric hospitalizations between living patients with and without severe mental illness over time.

Time Interval	No Severe Mental Illness			Severe Mental Illness			*P*
	*Patients*	*Admits*	*Mean/SD*	*Patients*	*Admits*	*Mean/SD*	
*Pre-Stroke*							
<1 year	422	424	1.00 ±1.33	100	147	1.47 ± 0.51	0.0004
*Post Stroke*							
≤ 30 days	390	108	0.27 ± 0.37	95	49	0.52 ± 0.62	0.0310
31 to 90 days	371	161	0.43 ± 0.59	93	50	0.53 ± 0.62	0.0491
91 to 364 days	321	291	0.91 ± 1.41	87	104	1.19 ± 1.64	0.0017
< 1 year (Subtotal)	321	560	1.74 ± 1.86	87	203	2.33 ± 2.46	0.0004
1 to < 2 years	274	252	0.89 ± 1.19	79	78	0.97 ± 1.55	0.2934
2 to < 3 years	249	205	0.83 ± 1.43	74	58	0.78 ± 1.21	0.9600
3 to < 4 years	214	174	0.81 ± 1.23	69	41	0.59 ± 1.24	0.0582
4 to < 5 years	166	177	1.06 ± 1.79	59	40	0.67 ± 1.36	0.0413

The patient column represents the number of individuals who remained alive during the entire time interval. The admits column represents the total number of non-psychiatric hospital admissions during each time interval. The mean/SD column is a calculation of the average number of admissions among the living patients during the time interval. The p-value was calculated using the pooled t-test method.

Tables 4 and 5 summarizes the results of the multiple Poisson regression by transforming beta coefficients from the regression into relative risk calculations, which allow for interpretation and testing of the hypothesis that SMI is associated with increased non-psychiatric hospitalizations, after adjustment by covariates. PTSD exhibited a confounding relationship with SMI during the time intervals of 91 to 364 days and 3 to < 4 years post-stroke. History of depression was also confounding with SMI during the time intervals of ≤ 30 days, 91 to 364 days, and 1 to < 2 years post-stroke. No other independent variables exhibited a confounding relationship with SMI. After adjusting for covariates, there are no longer significant differences in the risk for non-psychiatric hospitalizations between patients with and without SMI at any time period after stroke.

**Table 4:** Poisson multiple regression assessing the association of SMI with non-psychiatric hospitalization over time-intervals from ≤ 30 Days through 2-years post-stroke.

	≤ 30 Days	31to 90 Days	91 to 364 Days	1 to < 2 Years
History of Severe Mental Illness	1.78 [0.89–2.49]	.83[.54–1.21]	1.34 [.91–1.98]	.85 [.54–1.29]
Age 45–64 years (ref 18–44)	1.20 [.71–2.03]	1.13 [.66–1.95]	.69 [.39–1.23]	1.10 [.55–2.22]
Age 65–74 years (ref 18–44)	.95 [.52–1.75]	.81 [.44–1.47]	.86 [.45–1.67]	.90 [.42–1.94]
Age > 75 years (ref 18–44)	1.12 [.61–2.03]	1.18 [.65–2.15]	.91 [.47–1.75]	1.35 [.63–2.82]
Race (ref White)	1.23 [.91–1.67]	1.03 [.78–1.34]	.66 [.46–.91]**	.78 [.56–1.09]
Elixhauser Index	1.09 [.96–1.22]	1.04 [.94–1.09]	1.21 [1.12–1.33]***	1.13 [1.02–1.24]*
Pre-Stroke Hospitalizations	.98 [.95–1.04]	.99 [.95–1.03]	1.01 [.96–1.10]	1.06 [1.01–1.11]**
History of PTSD	.82 [.59–1.13]	.78 [.58–1.05]	removed	1.03 [.71–1.50]
History of Depression	removed	removed	removed	removed
History of Substance Abuse	.99 [.68–1.44]	.97 [.67–1.39]	.98 [.63–1.51]	.50 [.30–.81]*
History of Hypertension	.91 [.61–1.36]	.90 [.62–1.30]	.79 [.50–1.28]	.98 [.59–1.61]
History of Diabetes	.93 [.69–1.24]	1.13 [.87–1.46]	.95 [.66–1.35]	1.39 [.98–1.97]
History of Peripheral Vascular Disease	.94 [.72–1.54]	1.11 [.79–1.55]	.75 [.47–1.18]	.73 [.46–1.14]
Income	1.00 [1.00–1.00]	1.00 [1.0–1.0]	1.00 [1.0–1.0]	1.00 [1.0–1.0]
Insurance Status	.96 [.72–1.29]	1.07 [.82–1.39]	1.03 [.72–1.45]	.87 [.62–1.23]
Marital Status	.89 [.68–1.18]	.99 [.77–1.29]	.89 [.63–1.24]	.84 [.60–1.15]
Distance from VA to Home	.99 [.99–1.01]	.99 [.98–1.0]	.99 [.98–1.01]	.99 [.97–1.00]†
Discharged on a Statin	1.03 [.78–1.36]	.90 [.71–1.17]	.98 [.68–1.37]	.63 [.45–.88]**
Antithrombotic at Discharge	.62 [.50–.74]*	.81 [.59–1.02] †	.90 [.51–1.56]	.69 [.51–1.09]
Assessed for Rehabilitation	1.18 [.87–1.64]	.91 [.67–1.24]	.83 [.54–1.24]	.71 [.48–1.07]
Length of Hospital Stay	1.02 [.77–1.34]	1.01 [.78–1.32]	1.19 [1.07–1.30]*	1.11 [.90–1.31]
Observations Used (N)	493	454	437	397
Value/df	1.04	1.06	.86	.86
Null Scale Deviance	537.0	564.6	362.8	332.8
Scaled Deviance	468.7	490.3	356.7	322.9
AIC (smaller is better)	841.0	930.2	1257.2	1199.2

The values in the table are the Relative Risks [95% Confidence Intervals] where statistical significance is denoted by ***p<0.001, **p<0.01, *p<0.05, †p<0.10.

Cells indicating “removed” were not considered in the model due to multicollinearity with SMI. Risk for increased hospitalization was calculated by taking the estimated Poisson regression coefficient (β) for each variable and transforming it by e^β^ [exp*confidence interval] of each independent variable for the model. The ratio of value to degrees of freedom (value/df) when close to 1.0 indicates adequate fit. A null model was fit with only SMI and no covariates and its scaled null deviance indicates the total variance available for explanation. The scaled deviance indicates the amount of variance explained by the model.

**Table 5:** Poisson multiple regression assessing the association of SMI with non-psychiatric hospitalization over time-intervals from 2-years through 5-years post-stroke.

	2 to < 3 Years	3 to < 4 Years	4 to < 5 Years
History of Severe Mental Illness	1.04 [.60–1.78]	.81 [.40–1.68]	.72 [.31–1.68]
Age 45–64 years (ref 18–44)	3.57 [.99–12.68]	2.43 [.68–8.91]	1.05 [.39–2.77]
Age 65–74 years (ref 18–44)	2.46 [1.05–9.31]	1.09 [.26–4.52]	.69 [.22–2.13]
Age > 75 years (ref 18–44)	3.91 [.98–14.61]	3.03 [.78–11.75]	3.42 [.40–3.93]
Race (ref White)	.83 [.94–1.22]	1.28 [.77–2.15]	1.40 [.80–2.48]
Elixhauser Index	1.11 [1.03–1.26]*	1.18 [1.04–1.35]**	1.16 [1.02–1.36]*
Pre-Stroke Hospitalizations	.99 [.93–1.06]	1.07 [.99–1.16]†	1.09 [1.01–1.17]*
History of PTSD	1.13[.73–1.80]	removed	.71 [.37–1.35]
History of Depression	.90 [.53–1.51]	.43 [.20–.92]*	.58 [.27–1.24]
History of Substance Abuse	.83 [.46–1.42]	.49 [.25–.98]*	.74 [.36–1.49]
History of Hypertension	.72 [.41–1.25]	1.29 [.58–2.90]	2.41 [.87–6.75]
History of Diabetes	1.04 [.69–1.56]	.70 [.41–1.19]	.93 [.52–1.63]
History of Peripheral Vascular Disease	1.01 [.59–1.69]	.74 [.37–1.47]	1.05 [.53–2.11]
Income	1.00 [1.0–1.0]	1.00 [1.0–1.0]	1.00 [1.0–1.0]
Insurance Status	1.47 [.99–2.22]†	.86 [.51–1.45]	.83 [.46–1.49]
Marital Status	1.04 [.69–1.54]	1.10 [.64–1.82]	.81 [.48–1.37]
Distance from VA to Home	.99 [.98–1.01]	1.01 [.98–1.01	.99 [.98–1.01]
Discharged on a Statin	.96 [.64–1.43]	1.11 [.68–1.84]	.63 [.37–1.04]
Antithrombotic at Discharge	.63 [.49–3.49]	1.23 [.46–3.29]	1.40 [.49–3.95]
Assessed for Rehabilitation	1.16 [.75–1.79]	1.09 [.63–1.90]	1.32 [.74–2.34]
Length of Hospital Stay	1.04 [.70–1.49]	1.02 [.69–1.50]	1.01 [.70–1.48]
Observations Used (N)	338	300	259
Value/df	.86	.72	.77
Null Scale Deviance	284.0	212.9	187.9
Scaled Deviance	272.7	198.7	180.9
AIC (smaller is better)	874.5	716.7	590.2

The values in the table are the Relative Risks [95% Confidence Intervals] where statistical significance is denoted by ***p<0.001, **p<0.01, *p<0.05, †p<0.10.

Cells indicating “removed” were not considered in the model due to multicollinearity with SMI. Risk for increased hospitalization was calculated by taking the estimated Poisson regression coefficient (β) for each variable and transforming it by e^β^ [exp*confidence interval] of each independent variable for the model. The ratio of value to degrees of freedom (value/df) when close to 1.0 indicates adequate fit. A null model was fit with only SMI and no covariates and its scaled null deviance indicates the total variance available for explanation. The scaled deviance indicates the amount of variance explained by the model.

While history of SMI was not associated with post-stroke non-psychiatric hospitalizations after adjustment, a number of other independent variables were signficantly associated with non-psychiatric hospitalizations. Patients discharged on antithrombotic medications had a lower risk for re-admission (RR = .62; 95% CI: .50–.74) for a stroke-related complication within 30-days of index stroke discharge compared to patients not discharged on antithrombotics. During the time period 91 to 364 days post-stroke discharge minority patients (RR = 0.66; 95% CI: .46–.91) had signficantly lower risk for non-psychiatric hospitalizations compared to white patients, while patients with higher Elixhauser co-morbidity index scores (RR = 1.21; 95% CI: 1.12–1.33) were at greater risk for non-psychiatric hospitalizations. Additionally, during the time period 91 to 364 days the longer the initial stroke hospital length stay the greater risk for non-psychiatric hospitalizations (RR = 1.19; 95% CI: 1.07–1.30). During the time period 1 to < 2 years post-stroke patients with higher Elixhauser co-morbidity index scores (RR = 1.13; 95% CI: 1.02–1.24) and patients with more hospitalizations in the year preceding index stoke (RR = 1.06; 95% CI: 1.01–1.11) were at signficantly greater risk for non-psychiatric hospitalizations, while patients with a history of substance abuse (RR = 0.50; 95% CI: 0.30–0.81) had lower risk for non-psychiatric hsopitalization. Additionally, patients discharged on statin medications (RR = .63; 95% CI: .45–.88] also had lower risk of non-psychiatric hospitalizaiton. During the time period 2 to < 3 years post-stroke only patients with higer Elixhauser co-morbidity index (RR = 1.11; 95% CI: 1.03–1.26) were at signficantly increased risk for non-psychiatric hospitalization. During the time period 3 to < 4 years post-stroke patients with higher Elixhauser co-morbidity index scores (RR = 1.18; 95% CI: 1.04–1.35) were at significantly greater risk for non-psychiatric hospitalizations, while patients with at history of depression (RR = .43; 95% CI: .20–.92) and a history of substance abuse (RR = .49; 95% CI: .25–.98) were at lower risk for non-psychiatric hospitalizations. Lastly, during the time period of 4 to < 5 years post-stroke, patients with higher Elixhauser co-morbidity index scores (RR = 1.16; 95% CI: 1.02–1.36) and patients with more hospitalizations in the year preceding index stroke (RR = 1.09; 95% CI: 1.01–1.17) were at signficantly greater risk for non-psychiatric hospitalizations.

Tables 4 and 5 present results using an approach of fitting a full model and using data reduction for collinear variables to test the hypothesis that SMI is associated with non-psychiatric hospitalizations. As a final measure to ensure accurate interpretation of results, variables except for SMI were removed from the model if α > .5 to achieve greater parsimony. For instance, in the time period of <30 days only the variables history of SMI, race, history of PTSD, marital status, income, distance from VA to home, antithrombotic at discharge, and assessed for rehab were retained in the model, while ten other variables were excluded because they had α > .5. This procedure was repeated for each of the time periods yielding no meaningfully divergent results from a full model presented in Tables 4 and 5. The same variables retained statistical significance at each time period with similar relative risks for non-psychiatric hospitalization. None of the parsimonious models demonstrated history of SMI was significantly associated with increased risk of non-psychiatric hospitalization after controlling for covariates.

## Discussion

The aim of this study was to determine the relationship between SMI and non-SMI and the risk for non-psychiatric hospitalization in the five years after index stroke. In unadjusted descriptive analyses, patients with a history of SMI had significantly higher mean non-psychiatric hospitalizations in the year prior to index stroke and over the course of the entire first year post-stroke compared to patients with no SMI comorbidity. In Poisson regression analysis after adjusting for a broad set of patient characteristics, differences in non-psychiatric hospitalizations between the two groups were no longer significant. Patient characteristics included in the regression analysis were age, marital status, income, insurance status, proximity to hospital, physical co-morbidity, acute mental health history, hospital length of stay at index stroke, and quality of inpatient stroke treatment. This analysis highlights that patients with SMI are medically complex with multiple risk factors for hospitalization, including significantly higher levels of physical co-morbidity. Patients with this type of profile, notwithstanding mental illness, are likely to have increased non-psychiatric hospitalizations. Because individuals with SMI have extensive co-morbidity [5, 6], tend to delay care [36], engage in risky health behaviors [8], and do not effectively adhere to prescribed treatment [37], their physical health and hospitalization rates are influenced by their mental health status.

Our findings differ with several studies examining the association between hospital admissions and mental illness in the general population. Saravay and colleagues [38] found that those with post-stroke depression averaged twice as many readmissions and spent twice as many days re-hospitalized over a 4-year period. Similarly, Borckardt and colleagues [39] found that psychiatrically involved outpatients had higher average readmissions (mean = 1.6) compared to non-psychiatric outpatients (mean = 1.34) over one year. However, both studies only report unadjusted readmissions and do not utilize regression techniques to account for the effects of other patient characteristics on hospitalizations.

In our sample, 43.3% of veterans had health insurance coverage with Medicare or Medicaid or both. Patients with SMI were significantly more likely (70%) to have only VHA benefits compared to those without SMI (54%). Those veterans with Medicare or Medicaid are likely to have received a portion of their care outside of the VHA system. Since we did not have access to data describing non-VHA care we are unable to account for the full spectrum of care delivered to stroke patients in our sample. However, it is more likely that stroke survivors with SMI received more of their care in the VHA system compared to those without SMI. Nevertheless, insurance status was not a significant predictor of hospitalizations in our sample.

It is possible that the results obtained by the regression analysis reflect the success of longstanding effort to integrate physical and mental health care in the VHA. Hankin and colleagues [40] indicate that the VHA health care system has been intentionally designed to effectively manage patients with mental illness. In the mid-1990s, the VHA health care system launched an extensive reengineering process with the intent of creating greater integration of primary care with mental health services. The VHA’s design process recognized and attempted to correct system-level factors that contribute to poor health outcomes such as poor access to care, and ubiquitous segregation of medical and mental healthcare leading to disjointed, uncoordinated, and poor quality care [12, 41]. Several subsequent studies evaluating the effectiveness of the VHAs care integration have emerged over the last decade, all of which demonstrate substantial improvement in mental health quality of care indicators [42–44]. In the general population (non-VHA), suboptimal management of comorbidities is widely reported among patients with SMI, as are unfavorable disparities in morbidity and mortality [45]. In VHA systems of care, individuals with SMI die 13.8 years earlier than those without SMI, contrasted with non-VHA systems of care where individuals with SMI die 25 years earlier [46]. In a comparison of the quality of medical care in VHA and non-VHA settings between 1990 and 2009, the care delivered in VHA showed greater adherence to accepted processes of care, greater rates of evidence-based pharmaceutical therapy, greater mental and primary care provider co-location, greater collaboration on diagnostic and treatment plans, improved quality monitoring and better health outcomes [45, 47]. These findings suggest that the VHA’s model for integrated medical and mental health care reduces disparities in years of lost life and could partially explain the findings of our study. Future research should examine the impact of the integrated nature of VHA health care among patients with SMI to determine the extent to which coordinated care confers benefits to this population, and whether the VHA model could provide lessons to improve care for the SMI in health care reform.

It is notable that in the time period of within 30-days post-stroke, both SMI and non-SMI patients who were discharged on antithrombotic medications had significantly lower risk for stroke-related readmission. Blood clots complicate recovery from stroke [13] and antithrombotic medications reduce the formation of such clots. Our findings validate the use of antithrombotic agents to reduce unnecessary future morbidity, which may have relevance and implications in the current landscape of health care reform [48]. Additionally, patients with higher Elixhauser comorbidity index scores were at greater risk of non-psychiatric hospitalization after 91 days post-stroke, which is consistent with the results of several studies [49, 50]. Our finding validates previous work indicating that physical comorbidity increases risk for hospitalizations after surviving a stroke.

There are several limitations of our study that may have implications on the interpretation and generalizability of results. First, caution should be used when generalizing results beyond the VHA because of the unique sociodemographic and health profiles of the veteran population and the integrated nature of mental health care within the VHA. Second, the study utilized administrative data that, as we have noted, has limitations. Third, as mentioned previously, our data set does not contain health care utilization outside of the VHA system. Since veterans without SMI were more likely to have Medicare, Medicaid or other insurances, they would be more likely than the SMI to have utilized health services that remain unmeasured in this study. Further, since the VHA does not provide emergency department services for acute stroke, patients being transported to the hospital in response to a 911 call will be taken to a non-VHA hospital. We assume that veterans without health insurance, and particularly those with SMI, who require hospitalization will be transferred as soon as possible to the VHA. Consequently, examining non-VHA care might yield different results regarding the relationship between SMI and non-psychiatric hospitalizations. Fourth, several additional relevant variables were not available for our analysis. For instance, there were no direct measures of stroke severity based on clinical measures, rather we relied on index stroke hospital length of stay as a proxy for severity. Data on lifestyle factors such as obesity, alcohol use, smoking, and physical activity were also not available. Additionally, antipsychotic medication usage was not considered in regression models and may have provided valuable insight into the results. These factors should be assessed in future studies.

Strengths of the study are 1) the VHA system is one of the largest integrated and standardized health systems in the United States and regular audits conducted by clinical specialists are in place to cross-check diagnosis codes with more robust patient chart review [51]. Moreover, research from as early as 1998 has demonstrated adequate reliability for demographic variables (kappa = 0.92), diagnosis variables (kappa = 0.39–1.0) and cohort identification in administrative data in the VHA system [52, 53]. 2) This longitudinal VHA database provides a good opportunity to examine post stroke utilization for the SMI, in part because a large percentage of this population has no other source of health coverage.

## Conclusion

The severely mentally ill present a challenging and costly patient population for health systems. Although previous studies have examined hospitalizations among stroke survivors, limited data are available on the burden of SMI on post-stroke non-psychiatric hospitalizations. Unadjusted results highlight differences between the SMI and non-SMI stroke survivors, with the SMI experiencing more non-psychiatric hospitalizations both prior to and up to one year after their initial stroke. After adjusting for numerous patient characteristics, we found that patients with SMI, who were receiving services in the VHA system, were not at higher risk for non-psychiatric hospitalization during any time period after their index stroke for up to five years, when compared to patients without SMI. These findings suggest that the integrated nature of VHA healthcare for patients with SMI may confer benefits to this high risk population through coordination between mental and physical health care. Because integrated services for the SMI are considered one of the strengths of the VHA, our findings are provocative, suggesting that the VHA model may be useful to other health systems as the country is challenged by an aging population living with SMI, stroke, and other chronic conditions. Additionally, the adjusted difference demonstrating that individuals discharged on antithrombotic medications were at lower risk of re-admission within 30 days highlight the importance of this quality indicator for effective stroke management, irrespective of mental illness.

## Supporting information

S1 Appendix SAS algorithms. Code identifying ICD-9 diagnosis, procedure, and inpatient pharmacy administration codes for stroke processes of care.
(DOCX)
