# Supplementary material for: Stroke survivors with severe mental illness: Are they at-risk for increased non-psychiatric hospitalizations?
Source: PLoS One. 2017 Aug 11;12(8):e0182330. doi: 10.1371/journal.pone.0182330 (PMC5553814; doi:10.1371/journal.pone.0182330)
Supplement: S1 Appendix — Code identifying ICD-9 diagnosis, procedure, and inpatient pharmacy administration codes for stroke processes of care. (DOCX) [file pone.0182330.s001.docx]

**Appendix 1**

The following are the algorithms created to identify stroke processes-of-care:

Antithrombotic therapy prescribed at discharge

IF [patient positive for drug class code CN103 (aspirin or dipyridamole)] OR [patient positive for drug class code BL100 (warfarin or heparin)] OR [patient positive for drug class code BL700 (clopidogrel or ticlopidine)] THEN [Yes, evidence of compliance] ELSE [No, evidence of compliance]

Assessed for rehabilitation services

IF [patient positive ICD-9 procedure code 9327 (stretching of muscle or tendon) OR 9330 (assisted exercise in pool) OR 9338 (combined physical therapy) OR 9319 (exercise unclassified) OR 9385 (vocational rehabilitation) OR 9389 (rehabilitation unclassified)] OR 9309 (physical therapy procedure) OR 9311 (assisting exercise) OR 9312 (other active musculoskeletal exercise) OR 9313 (resistive exercise) OR 9314 (training in joint movements) OR 9316 (mobilization of other joints) OR 9317 (other passive musculoskeletal exercise) OR 9455 (referral for vocational rehabilitation) THEN [Yes, evidence of compliance] ELSE [No, evidence of compliance]

Lipid management: patient discharged on statin medication

IF [patient positive for drug class code CV350 (simvastatin, lovastatin, or atovastatin) THEN [Yes, evidence of compliance] ELSE [No, evidence of compliance]
